# Thyroid function and urinary concentrations of iodine, selenium, and arsenic in vegans, lacto-ovo vegetarians and pescatarians

**DOI:** 10.1007/s00394-023-03218-5

**Published:** 2023-08-17

**Authors:** Sigrun Henjum, Synne Groufh-Jacobsen, Inger Aakre, Elin Lovise Folven Gjengedal, Mina Marthinsen Langfjord, Espen Heen, Veronika Sele, Maria Andersson

**Affiliations:** 1 Department of Nursing and Health Promotion, Faculty of Health Sciences, Oslo Metropolitan University, Kunnskapsveien 55, 2007 Kjeller, 0130 Oslo, Norway; 2 Department of Nutrition and Public Health, Faculty of Health and Sport Science, University of Agder, Universitetsveien 25, 4630 Kristiansand, Norway; 3 Department of Marine Toxicology, Institute of Marine Research, 5817 Bergen, Norway; 4 Faculty of Environmental Sciences and Natural Resource Management, Norwegian University of Life Sciences, 1432 Ås, Norway; 5 Institute of Health and Society, Medical Faculty, University of Oslo, Oslo, Norway; 6 Nutrition Research Unit, University Children’s Hospital Zurich, Steinwiesstrasse 75, 8032 Zurich, Switzerland

**Keywords:** Plant-based diet, Thyroid hormones, Thyroglobulin, Iodine, Urinary iodine, Selenium, Arsenic

## Abstract

**Purpose:**

Populations following a plant-based diet may be at particular risk of thyroid dysfunction due to low iodine and selenium intakes. The main purpose was to assess thyroid function and urinary concentration of iodine, selenium, and arsenic, in subjects following a vegan, lacto-ovo vegetarian, or pescatarian diet.

**Methods:**

In Norway, a country without mandatory dietary iodine fortification, 205 adults, following vegan (*n* = 115), lacto-ovo vegetarian (*n* = 55) and pescatarian diet (*n* = 35) were included. Thyroglobulin (Tg), thyroid-stimulating hormone (TSH), free triiodothyronine (fT3), free thyroxine (fT4), and serum anti-TPO (S-anti-TPO) were measured in a venous blood sample and concentrations of iodine (UIC), creatinine (UCC), selenium, and arsenic were measured from single spot urine samples.

**Results:**

Subclinical hypothyroidism (TSH > 4.0 mU/L) was observed in 3% of subjects. The overall median (p25, p75) Tg was 17 (9, 30) µg/L and vegans had higher Tg compared to pescatarians. Vegans not consuming iodine-containing supplements (*n* = 43) had higher Tg, than supplement users (*n* = 72), 27 (11, 44) vs. 16 (8, 25) µg/L and higher fT4, 16 (15, 17) vs. 15 (14, 17) pmol/L, respectively. The overall median UIC was 57 (28, 130) µg/L, all dietary groups had median UIC below WHO thresholds. Median urinary selenium and arsenic concentration was 13 (6, 22) and 3 (2, 8) µg/L, respectively.

**Conclusion:**

The prevalence of subclinical hypothyroidism was low and fT4 and fT3 were within the normal range for all dietary groups. Vegans had significantly increased Tg compared to pescatarians.

**Supplementary Information:**

The online version contains supplementary material available at 10.1007/s00394-023-03218-5.

## Introduction

Iodine is an essential micronutrient needed for the synthesis of thyroid hormones triiodothyronine (T3) and thyroxine (T4) and adequate production is required for growth, development, and regulation of the metabolism [1]. Mild to moderate iodine deficiency is widespread among adults in Europe [2], including Norway [3, 4]. Globally, the iodization of table salt is the main strategy to prevent iodine deficiency disorders and iodized salt is the primary dietary source of iodine in countries with mandatory iodine fortification policy [5]. Norway has not implemented salt iodization and low iodine intake has been reported among women of fertile age, pregnant and breastfeeding women, infants who are exclusively breastfed, elderly, vegans, and immigrants [4]. In Norway, animal source foods such as cow’s milk and yoghurt, eggs (due to iodine fortification of fodder), and marine fish are the main dietary iodine sources. WHO recommend assessment of median urinary iodine concentration (UIC) as an indicator for iodine status in a population [5]. In addition to UIC, thyroglobulin (Tg) can also serve as a marker of iodine deficiency and altered thyroid activity. When iodine intakes decrease, iodine stores in the thyroid gland diminish [6]. Tg, a protein synthesized by the thyroid cells, increases thyroid activity to maintain thyroid hormone concentrations within the normal range [7, 8].

People adhering to a plant-based diet can be categorized as vegans (no intake of animal source foods) lacto-ovo vegetarian (intake of milk/dairy products, cheese and/or eggs) or pescatarian (intake of milk/dairy products, cheese and/or eggs, in addition to fish/shellfish/fish products). Plant-based diets are typically associated with low iodine intakes compared to omnivore diets [9], unless iodine-containing supplements or microalgae is consumed. The iodine content in macroalgae may, however, vary widely both between and within species, and some products contain excessive amounts of iodine [10], and may be an unreliable iodine source [11]. Plant-based milk and dairy products have low iodine content, unless they are fortified with iodine [12, 13].

The synthesis of thyroid hormones is catalyzed by thyroid peroxidase (TPO) which uses selenium as a cofactor. Selenium is an essential element that is required for selenocysteine synthesis and production of selenoproteins [14]. Animal source foods such as dairy products, meat and seafoods have high selenium concentration, and for plant-foods, the concentration of selenium depend on the selenium level in soil. Lower selenium status has been reported in vegetarians and vegans as compared to omnivores in epidemiological studies [15]. Urine is the main route of excretion of selenium, mainly in the form of selenosugar [16] and have been considered as a useful marker of recent selenium intake [17] and a biomarker of selenium status of a population [18].

Inorganic arsenic has been found to inhibit TPO in vitro and arsenic exposure has been associated with thyroid dysfunction. Arsenic is an element that can exist in various chemical forms, with inorganic arsenic classified as carcinogenic whereas the organic form arsenobetaine is regarded non-toxic, in addition to many chemical forms not yet fully characterized for their toxicity [19]. In a Norwegian randomized controlled trial, ingestion of seafood rich in total arsenic was associated with an increase in thyroid-stimulating hormone (TSH) [20]. Most arsenic compounds present in food items are excreted in urine with a half time generally of a few days, and therefore, measurement of total urinary arsenic has been used as a biomarker of arsenic exposure [21, 22].

Since the most important dietary iodine sources in Norway are found in animal source foods, individuals following a plant-based diet are at particular risk of iodine deficiency [23], but the impact on thyroid function is uncertain. Thus, the objective of this study is to assess thyroid function and urinary iodine, creatinine, selenium, and arsenic concentration in subjects adhering to a vegan, lacto-ovo vegetarian, and pescatarian diet.

## Materials and methods

### Subjects and study design

Recruitment of participants in this cross-sectional study was conducted through convenience sampling and snowball sampling from September to November 2019, in the Oslo area in Norway. Participants were recruited through social media in closed Facebook groups and in online vegan and vegetarian forums. Details about the recruitment method and inclusion criteria have previously been described [23]. Study inclusion criteria were: (1) consumption of a vegan, lacto-ovo vegetarian or pescatarian diet for a minimum of 6 months; (2) age 18 years or older; (3) not currently pregnant or lactating; (4) no current use of thyroid medication.

We recruited 236 participants, and 29 people did not meet the inclusion criteria. In addition, two participants were excluded from the data analysis because of thyroid medication use and occasional meat consumption. Thus, the final sample consisted of 205 subjects, of which 115 vegans, 55 lacto-ovo vegetarians and 35 pescatarians. Participants who had provided study consent filled out an electronic questionnaire which consisted of background characteristics (age, anthropometric measures (height and weight), marital status, occupational status, educational level, smoking habits, country of birth, language, duration of adherence to vegan/lacto-ovo vegetarian/pescatarian diet) and foods included in the diet the previous 6 months (used to categorize participants into the different diet groups). The participants also conducted a 24-h dietary recall and a food frequency questionnaire (FFQ) for assessment of dietary intakes the previous 4 weeks (dietary iodine intakes are not presented in this paper, as these data have previously been published [23].

### Assessment of dietary supplement use and macroalgae use

Iodine supplement use was assessed both by 24 h and habitual intake the previous 4 weeks (FFQ). The participants reported the name of the supplement, brand and amount used during the previous 24 h. By habitual intake, supplement consumption was reported as frequency per week, e.g., if a supplement contained 150 µg, and if taken four times a week, the contribution was estimated to be (50 µg × 4/7) 86 µg/day. Selenium-containing dietary supplements were also obtained from a FFQ in the electronic questionnaire in the same way as for iodine. For assessment of consumption of macroalgae, a dichotomous variable (no/yes) was used, if yes, participants reported type and amount (gram) used the previous 24 h and the habitual use the past 4 weeks. The types of macroalgae reported were Sugar kelp (*Saccharina latissimi*), Bladder wrack (*Fucus vesiculosus*), Wakame (*Undaria pinnatifida*), Kombu (*Laminaria japonica and Saccharina japonica*), Dulse (*Palmaria palmata*) and Laver (*Porphyra* spp.), with more details described in previous work [23].

### Determination of concentrations of elements in urine

All participants (*n* = 205) provided one non-fasting spot urine sample collected non-fasted at random times throughout the day, in a labeled 100 mL Vacuette^®^ urine beaker (Greiner Bio-One, Kremsmünster, Austria). A subsample of urine was transferred into a 9.5 mL Vacuette^®^ urine tube (Greiner Bio-One, Kremsmünster, Austria) and immediately put to storage at 2–4 °C before freezing at – 80 °C until analyses. The urine concentrations of iodine, selenium, and arsenic were measured at the Norwegian University of Life Science, Faculty of Environmental Science and Natural Resource Management. The analysis was performed using an alkaline sample preparation and subsequent quantitative determination using Inductively Coupled Plasma Mass Spectrometry ICP-MS [23]. An aliquot of 1 mL of urine was transferred into 15 mL pp centrifuge tubes (Sarstedt, Nümbrecht, Germany) by means of 100–5000 µL electronic pipette (Biohit, Helsinki, Finland) and diluted to 10 mL adding an alkaline solution (BENT), containing 4% (weight (w)/volume(V) 1-Butanol and 0.1% (w/V) H_4_EDTA, 2% (w/V) NH_4_OH, and 0.1% (w/V) TritonTM X-100. Method blank samples and samples of standard reference material (SRM) were prepared following the same procedures. Reagent of analytical grade or better and deionized water (18 MΩ) were used throughout. The samples were analyzed for iodine, selenium, and arsenic concentration using an Agilent 8800 ICP-QQQ (Triple Quadruple Inductively Coupled Plasma Mass Spectrometer, Agilent Technologies, Hachioji, Japan) using oxygen as a reaction gas. The concentration of iodine was determined by measuring ^127^I isotope. Simultaneously, the concentration of selenium in urine was determined on by measuring the mass shift from 78 to 94, while arsenic in urine was determined using a mass shift from 75 to 91. The limits of detection (LOD) and limits of quantification (LOQ) were calculated by multiplying the standard deviation of the method blank samples (*n* = 10) by three and ten, respectively. The obtained LOD and LOQ were 0.3 µg/L and 0.92 µg/L for iodine, 0.06 µg/L and 0.2 µg/L for selenium and 0.01 µg/L and 0.05 µg/L for arsenic, respectively. To check for method accuracy, two reference materials of urine (Sero AS, Billingstad, Norway) were analyzed; each with value assignment established in accordance with the Essential Requirements of the IVD Directive1 98/79/EC, and the ISO 17511 International standard [24]. The results were within the recommended values issued for the SeronormTM Trace Elements Urine L-1 and SeronormTM Trace Elements Urine L-2. The measurement repeatability was investigated in two different urine samples, one with visible precipitates and the other one completely transparent (each one with *n* = 5). The relative standard deviation (RSD) was 2.3% for iodine, 1.3% for selenium (up to 6.5% for samples with low concentrations), and < 1.0% for arsenic. UCC was measured at Fürst Medical Laboratory, Oslo, Norway.

### Definitions

Epidemiological criteria defined by WHO [5] for median UIC (not creatinine adjusted) was used to assess population iodine status; UIC < 20 µg/L severe iodine deficiency; UIC < 50 µg/L moderate iodine deficiency; UIC < 100 µg/L mild iodine deficiency; UIC in the range 100–199 µg/L adequate iodine intake; UIC in the range 200–299 µg/L more than adequate iodine intake; UIC > 300 µg/L excessive iodine intake. There is no established reference value for evaluating selenium or arsenic status based on urinary concentration. Thus, selenium and arsenic urinary concentrations were compared against measures of urinary selenium and arsenic in healthy populations [25, 26]. The urinary concentration of selenium range from 10 to 90 μg/L [26]. Regarding arsenic, in a European reference population (with no occupational exposure, no seafood consumption and drinking water concentration below 10 µg/L), mean concentrations of urinary inorganic arsenic and related metabolites are around 5–6 µg/L [27].

We adjusted UIC for creatinine for each participant by the following equation [28, 29]:

### Analysis of thyroid function markers

We collected a non-fasting venous blood sample and measured serum thyroglobulin (Tg), serum thyroid-stimulating hormone (S-TSH), free triiodothyronine (S-fT3), free thyroxine (S-fT4) and serum anti-TPO (S-anti-TPO). S-Tg was measured in duplicate using a sandwich serum Tg enzyme-linked immunosorbent assay (ELISA) [30]. Liquicheck Tumor Marker Control (Bio-Rad Laboratories AG, Cressier, Switzerland; LOT. 24000) was used as standard. We used laboratory-specific external quality control samples and the presented data complied with the defined criteria. The limit of detection for serum Tg assay was 2.3 µg/L [30]. For S-Tg concentrations below the limit of detection (LOD) (2.3 µg/L), we used a number generator and assigned a random value between 0.1 and 2.3 µg/L. S-TSH, S-fT4 and S-fT3 were analyzed at Fürst Medical Laboratory, Oslo, Norway using Advia Centaur XPT-instruments (Siemens Healthineers, City, Country).

The reference values from Fürst medical laboratory (Oslo, Norway) were used to evaluate S-TSH, S-fT3, S-fT4 and S-Anti-TPO levels. S-TSH reference range 0.20–4.0 mU/L (> 19 years of age); S-fT3 reference range 3.5–6.5 pmol/L; S-fT4 reference range 11.0–23.0 pmol/L and S-Anti-TPO a cutoff > 100 kU/L were used. We defined thyroid dysfunction as outlined in Supplemental Table 1. No reference values are available in adults for the used Tg assay.

### Statistics

IBM SPSS versions 25, 27 and 29 (IBM Corp., Armonk, NY, USA) were used for statistical analysis. Normality of the data was checked using visual evaluation of the Q–Q plots and histograms. Normally distributed data were presented as mean ± standard deviation (SD) and non-normally distributed data as median and the 25th and 75th percentiles (p25–p75) in tables. Cross-tabulation with Chi-square test was used to test differences between the dietary groups at nominal level; gender (male, female) and supplement use (yes/no). Kruskal–Wallis test with correction for multiple comparison was used to test for difference between the dietary groups, and difference in the Bonferroni post hoc test is indicated with equal superscripts. *p* value < 0.05 was used as significance value throughout.

Multiple linear regression analysis was used to examine the association between vegan dietary practice with Tg levels. Before performing a multivariate-adjusted analysis, univariate regression analysis was performed to examine if there was an association between the independent variable (vegan diet coded as dummy variable with pescatarian as control group) with the Tg levels (dependent). The independent variables (age, gender, smoking, supplement use, and years of dietary practice) that were significantly associated with the Tg levels in the univariate regression analysis (significance level *p* value < 0·005) were included in the multiple regression analysis. Two cases were identified with standardized residuals above 3. Sensitivity analysis was performed with and without the outliers. The final model was adjusted for age (years, continuous) and gender (male ref).

## Results

The characteristics of the study population (*n* = 205) are shown in Table 1. Gender, diet duration and habitual use of iodine and selenium supplements differed between the diet groups, while characteristics as age, body mass index (BMI), educational level, smoking status, 24-h iodine supplement use and macroalgae use (habitual use), did not differ between the diet groups.

**Table 1:** Background characteristics of vegans (*n* = 115), lacto-ovo vegetarians (*n* = 55) and pescatarians (*n* = 35) in Norway

	All (*n* = 205)	Vegans (*n* = 115)	Lacto-ovo vegetarians (*n* = 55)	Pescatarians (*n* = 35)	*p* value^b^
Gender					**0.010**
Females, *n* (%)	148 (72.2)	74 (64.3)^c^	43 (78.2)^c,d^	31 (88.6)^d^	
Males, *n* (%)	57 (27.8)	41 (35.7)^c^	12 (21.8)^c,d^	4 (11.4)^d^	
Age, years^a^	30.4 ± 9.1	31.1 ± 8.7	30.3 ± 10.4	28.5 ± 7.8	0.186
Body mass index, kg/m^2a^	23.2 ± 3.5	23.0 ± 2.9	23.6 ± 4.5	23.2 ± 3.1	0.969
Educational level					0.684
< 12 years, *n* (%)	6 (2.9)	3 (2.6)	1 (1.8)	2 (5.7)	
12 years, *n* (%)	36 (17.6)	22 (19.1)	11 (20.0)	3 (8.6)	
1–4 years university, *n* (%)	163 (79.5)	90 (78.2)	43 (78.2)	30 (85.7)	
Smoking status, *n* (%)	20 (9.8)	12 (10.4)	6 (10.9)	2 (5.7)	0.778
Duration of diet, years^a^	4.7 ± 3.1	4.1 ± 2.7^c^	5.6 ± 3.4^c^	5.3 ± 3.6	**0.026**
Dietary supplement					
Iodine, 24 h, *n* (%)	100 (48.8)	57 (49.6)	25 (45.5)	18 (51.4)	0.723
Macroalgae habitually (4 weeks), *n* (%)	35 (17.1)	23 (20.0)	8 (14.5)	4 (11.4)	0.452
Selenium, habitually (4 weeks) *n* (%)	83 (40.5)	54 (46.9)^c^	21 (38.2)^c,d^	8 (22.8)^d^	**0.036**

^a^Mean value ± SD

^b^Kruskal–Wallis and Chi-square test to test for difference between the diet groups. Bold values mark statistically significant differences (*p* < 0.05)

^c,d^Diet groups with the same superscripts have proportions that differ significantly in the Bonferroni post hoc test (*p* < 0.05)

### Thyroid function markers

The overall median (p25, p75) Tg in the study population was 17 (9, 30) µg/L and vegans had higher median Tg compared to pescatarians (*p* = 0.028) Table 2. TSH was elevated (> 4.0 mU/L) in 3% of the study participants. The prevalence of subclinical hypothyroidism (elevated TSH) did not differ between vegans, lacto-ovo vegetarians, and pescatarians (*p* = 0.824). None of the participants had fT4 levels below < 11.0 pmol/L. We observed a significant, but weak, correlation between Tg and TSH (*p* = 0.041, *r*_s_ = 0.143), but no correlation between Tg and fT4 (*p* = 0.331, *r*_s_ = − 0.068). We observed no correlation between UIC and Tg (*p* = 0.073), TSH (*p* = 0.480), fT4 (*p* = 0.489) and anti-TPO (*p* = 0.540). Anti-TPO positivity (> 100 kU/L) was found in 18% (*n* = 204), of which four were vegans and five lacto-ovo vegetarians.

**Table 2:** Thyroid function markers in vegans (*n* = 115), lacto-ovo vegetarians (*n* = 55) and pescatarians (*n* = 35) in Norway

Thyroid function markers	All^a^ (*n* = 205)	Vegans ^a^ (*n* = 115)	Lacto-ovo vegetarians^a^ (*n* = 55)	Pescatarians^a^ (*n* = 35)	*p* value^b^	Reference
S-Tg (µg/L)	17 (9, 30)	18 (10, 36)^ab^	17 (10, 29)	11 (5, 20)^ab^	**0.028**	Not available
S-TSH (mIU/L)	1.4 (1.0, 1.9)	1.4 (1.0, 1.8)	1.4 (1.0, 1.8)	1.3 (0.9, 1.9)	0.824	0.2–4.0
S-fT3 (pmol/L)	5.1 (4.8, 5.5)	5.1 (4.8, 5.5)	5.2 (4.8, 5.6)	5.2 (5.4, 4.7)	0.874	3.5–6.5
S-fT4 (pmol/L)	15.8 (14.7, 17.3)	15.9 (14.8, 17.2)	16.0 (14.7, 17.6)	15.6 (14.4, 16.6)	0.419	
Anti-TPO (kU/L)	*n* (%)	*n* (%)	*n* (%)	*n* (%)	**0.013**	< 100
< 34	170 (82.9)	100 (87.0)	43 (78.2)	27 (77.1)		
> 34–100	29 (14.1)	14 (12.2)	7 (12.7)	8 (22.9)		
> 100	6 (2.9)	1 (0.9)	5 (9.1)	0 (0.0)		

*S-Tg* serum thyroglobulin, *S-TSH* serum thyroid-stimulating hormone, *S-fT3* serum free triiodothyronine, *S-fT4* serum free thyroxine, *anti-TPO* serum thyroid peroxidase antibodies

^a^Presented as median (p25, p75)

^b^Kruskal–Wallis test was used to test difference in Tg, S-TSH, S-fT3, S-fT4 and anti-TPO; *p* value < 0.05 was used as significance level are given in bold

^ab,cd,de^Diet groups with equal superscripts have proportions that differ significantly in the Bonferroni post hoc test (*p* < 0.05)

In vegans, median Tg was significantly higher in non-supplement users compared to supplement users (*p* = 0.004) (Table 3).

**Table 3:** Thyroglobulin and fT4 concentrations for iodine supplement users and non-iodine supplement users in Norwegian vegans, lacto-ovo vegetarians and pescatarians (*n* = 205)

	Iodine supplement users^a^	*n*	Min, max	Non-iodine supplement users^a^	*n*	Min, max	*p* value^b^
*S-Tg*							
Total	16 (8.8, 26.3)	113	1.0–139.90	18.2 (10.37)	92	0.30–235.10	0.628
Vegans	16.3 (8.0, 25.2)	72	1–140	26.8 (11.4, 44.0)	43	2–235	**0.004**
Lacto-ovo vegetarians	13.6 (9, 31)	29	2–126	20.4 (10.4, 29.7)	26	1–93	0.655
Pescatarians	16.9 (5.2, 32.1)	12	2–57	10.3 (5.1, 15.5)	23	0.3–40	0.144
*S-fT4*							
Total	16.1 (14.9, 17.3)	113	11.9–24.0	15.6 (14.2, 17.1)	92	11.6–21.8	0.400
Vegans	16.2 (14.9, 17.3)	72	12.2–24	15.1 (13.8, 16.7)	43	12–20.2	**0.045**
Lacto-ovo vegetarians	16.1 (14.7, 17.4)	29	12.6–18.4	15.8 (14.7, 18.0)	26	11.6–21.8	0.823
Pescatarians	15.5 (13.4, 16.4)	12	11.9–18.6	15.7 (14.4, 16.8)	23	11.7–19.7	0.676

^a^Presented as median (p25, p75)

^b^Kruskal–Wallis test was used to test the difference using Tg supplement users and non-users as continuous variables between the diet groups; *p* < 0.05 was used as a significance level are given in bold

Multiple linear regression analysis (Table 4) was used to examine the association between a diet without any iodine food sources (vegan dietary practice) and a diet including all iodine source (pescatarian dietary practice as control group) (independent variables) with Tg levels (dependent variable). Before performing a multivariate-adjusted analysis, univariate regression analysis (unadjusted) was performed to examine if there was an association between the independent variable (vegan diet coded as dummy variable with pescatarian as control group) with the Tg levels (dependent) (unadjusted). The independent variable was significantly associated with the Tg levels in the univariate regression analysis (unadjusted) (significance level *p* value < 0.005). The estimates were not influencing the regression model and were included in the final model. The final model was adjusted for age (years, continuous) and gender (male ref).

**Table 4:** Association between thyroglobulin concentration for people following a diet omitting all iodine sources (vegan diet) and with a diet including all iodine sources (pescatarian diet) (*n* = 150)

	Final model^a^		
	*β*^b^	95% CI^c^	*p* value
Constant (thyroglobulin)	38.48	23.04, 53.91	** < 0.001**
Vegan (pescatarian ref.)	− 12.83	− 23.08, − 2.57	**0.015**

Vegan = 0, pescatarian = 1

^a^The model is adjusted for age (years, continuous), gender (male ref)

^b^*β* standardized beta coefficients

^c^95% confidence interval for unstandardized *β*; statistically significant values < 0·05 are given in bold

### Urinary concentration of iodine, creatine-adjusted iodine concentration, selenium and arsenic

The median (p25, p75) UIC, creatine-adjusted UIC, selenium- and arsenic concentration in urine are presented in Table 5. The median UIC was 57 (28, 130) µg/L indicating overall inadequate iodine intake (UIC < 100 µg/L). Median creatine-adjusted UIC was 139 (63, 298) µg/day.

**Table 5:** Urinary concentration of iodine (UIC), creatine-adjusted UIC, urinary selenium, and urinary arsenic in vegans (*n* = 115), lacto-ovo vegetarians (*n* = 55) and pescatarians (*n* = 35) in Norway

Urinary concentration, µg/L	All^a^ (*n* = 205)	Vegans^a^ (*n* = 115)	Lacto-ovo vegetarians^a^ (*n* = 55)	Pescatarians^a^ (*n* = 35)	*p* value^b^
UIC total, µg/L	57 (28, 130)	43 (21, 120)^ab^	66 (34, 110)	95 (43, 160)^cd^	**0.030**
UIC, supplement users	63 (31, 160)	58 (29, 190)	69 (35, 105)	113 (46, 288)	0.410
UIC non-supplement users	48 (21, 100)	30 (12, 66)^ab^	69 (35, 105)	95 (37, 140)^cd^	**0.002**
Creatine-adjusted UIC total, µg/day^c^	139 (63, 298)	130 (59,130)	149 (64, 246)	189 (99, 343)	0.270
Creatine-adjusted UIC, supplement users^d^	181 (66, 319)	186 (64, 331)	141 (73, 240)	315 (65, 641)	
Creatine-adjusted UIC non-supplement users^e^	120 (60, 252)	93 (43, 218)	151 (62, 281)	121 (99, 296)	
Urinary selenium total, µg/L^f^	13 (6, 22)	11 (5, 22)	12 (7, 21)	15 (7, 36)	0.091
Urinary selenium, supplement users	11 (6, 22)	13 (5, 23)	12 (7, 21)	15 (7, 36)	0.573
Urinary selenium, non-supplement users	14 (6, 22)	10 (5, 20)	15 (7, 23)	17 (6, 39)	0.067
Urinary arsenic total, µg/L^g^	3 (2, 8)	3 (1, 5)^ab^	3 (2, 6)^ab^	21 (6, 57)^cd^	** < 0.001**

^a^Presented as median (p25, p75)

^b^Test for difference between the dietary groups: Kruskal–Wallis test with *p* value < 0.05 as significance value, marked as bold values

^ab,cd^Diet groups with different superscripts have proportions that differ significantly in the Bonferroni post hoc test (*p* < 0.05)

^c^Median creatine-adjusted UIC within the dietary groups

^d^Median creatine-adjusted UIC (µg/day) in iodine supplement user (24-h users)

^e^Creatine-adjusted UIC (µg/day) in non-iodine supplement or macroalgae users (habitual use)

^f^Urinary selenium concentration (µg/L) in selenium supplement user (habitual use) and non-supplement users

^g^Urinary arsenic, µg/L total and within the different diet groups

Vegans had lower median UIC compared to lacto-ovo vegetarians and pescatarians (*p* = 0.030), but when accounting for hydration (creatinine adjusted UIC), we observed no difference in UIC between the three diet groups (*p* = 0.270). Iodine supplement users had higher median UIC compared to non-iodine supplement users in all dietary groups [23]. For non-supplement users of iodine, vegans had lower median UIC compared to pescatarians (*p* = 0.002). None of the participants reported macroalgae consumption the previous 24 h. The overall median urinary concentration of arsenic was below < 15 µg/L. Vegans and lacto-ovo vegetarians had median arsenic concentrations < 15 µg/L and significantly higher concentrations (> 15 µg/L) were found in pescatarians (*p* < 0.001).

## Discussion

A normal thyroid gland can adapt to mild iodine deficiency and maintain thyroid hormone production [31]. The physiological response involves increased blood Tg concentration and higher thyroid activity [7]. Since there are no reference values for Tg in adults, we compared differences in Tg between the dietary groups. Vegans had higher Tg concentration compared to pescatarians, indicating that a low iodine intake increase thyroid activity and the thyroid production of Tg. Vegan non-supplement users had higher Tg compared to vegans consuming an iodine-containing supplements, consistent with other studies [7, 32]. Larger epidemiological studies have shown that prolonged thyroid stimulation associated with such an adaptation leads to thyroid growth, and during follicular cell proliferation, there is a tendency to mutations leading to multifocal autonomous growth and dysfunction [31, 33]. In populations with mild and moderate iodine deficiency, such multifocal autonomous thyroid function is a common cause of hyperthyroidism [34], particularly in elderly people, and the prevalence of thyroid enlargement and nodularity is higher than in iodine sufficient populations [31, 33]. In Denmark, profound effects of even minor differences in iodine intake level on the prevalence of goiter, nodules, and hyperthyroidism have been reported [35]. However, the incidence of multinodular toxic goiter and thyrotoxicosis decreased with improved iodine intake [36].

We found a low prevalence of thyroid dysfunction in our study and fT3, fT4 and TSH were within normal ranges. In the Adventist Health Study-2, there was no association between vegan diets and hypothyroidism [37]. However, in a more recent study focusing on a subsample of the Adventist Health Study-2, Tonstad found that those with elevated TSH (> 5 mUI/L) were more likely to be women following a vegan or vegetarian diet [38]. Leung et al. conducted a study in 140 vegetarians and vegans in USA and found TSH and FT4 concentrations in the normal range [39]. Although serum TSH was generally normal in 101 British vegans, mean TSH was 47% higher than for omnivores [40]. No thyroid function abnormalities were found in Swedish and Finnish vegans [41, 42].

Median UIC in the participating vegans, lacto-ovo-vegetarians and pescatarians indicated mild- to moderate iodine deficiency. Even in those using iodine supplements, UIC indicated inadequate iodine intake. It might be explained by the fact that iodine supplements with 150 µg iodine/day might not be sufficient in a people adhering to plant-based diets, or it can be challenges related to compliance with iodine supplements. In Norway, the iodine fortification of table salt is voluntary, and the permitted level of iodine is only 5 µg/g salt thus table salt is considered a negligible iodine source in the Norwegian diet [4]. Ovo-lacto vegetarians and pescatarian have iodine sources such as milk, dairy products, and eggs (fish for the pescatarian). Vegans have few iodine sources in their diet and are dependent on iodine supplements or intake of macroalgae to maintain an adequate iodine intake.

In our study, creatine-adjusted UIC, was higher than UIC. There are two factors that may contribute to this apparent disparity: with no meat intake in the study population, the total amount of creatinine destined for urine excretion (as creatinine) per 24 h may be less than in the general population from where the adjustment equation originates, inflating the creatine-adjusted UIC [43]. Second, a high urine volume may have diluted the iodine concentration in the spot sample reducing the expected UIC [6]. The WHO cutoff value for iodine deficiency is, however, not adjusted for creatinine, therefore, we compare UIC in our study with the WHO guidelines. WHO recommend use of spot urine as an easy and cost-efficient method for assessing iodine status in population groups. However, there is large inter- and intra-individual variation in UIC caused by differences in iodine intake as well as by large variation in fluid intake [5].

The total sample median urinary selenium concentration (13 µg/L) were below the reported levels in the Canadian Health Measures Survey [reference value (RV_95_)] of 120 µg/L [25] and in a Belgian population (RV_95_ of 62 µg/L) [44]. The levels are, however, within the same range as seen in a study of blood and urine of healthy unexposed subjects living in different regions of the United Kingdom, where a reference interval from 6 to 43 µg/L was set for selenium [45]. The findings of relatively low urinary selenium in our study are also comparable to a study by Fallon [46], which found low intakes and selenium in women of fertile age adhering to strict plant-based diets. Worldwide selenium intake varies considerably, with populations in Europe having relatively low selenium intake, compared to, e.g., North America and some parts of South America and Asia where selenium content of soil is high [47]. In this study, no difference was observed in selenium concentration between the different dietary groups. In Norway, fish was recently calculated to contribute with over 20% of the total mean intake of selenium for adults (general population) [48].

The median urinary concentrations of arsenic (3 µg/L) were low compared to a previous study from Norway where median total arsenic levels in urine were 102 µg/L, ranging from 8 to 859 µg/L [49]. Median urinary concentrations of arsenic in our study were also lower compared to the European reference populations where mean concentrations of urinary inorganic arsenic and related metabolites were 5–6 µg/L [27]. Total arsenic comprise of different arsenic species, whereas inorganic arsenic and the simple methylated forms are generally present in urine [27]. The toxicity of arsenic depends on the chemical form present, with inorganic arsenic being more toxic than organic arsenic compounds, although many compounds not yet fully characterized for their toxicity. Fish and seafood generally contain high levels of total arsenic, whereas the concentration of inorganic arsenic is usually low [27]. In most seafood, arsenic is mainly present in the form of arsenobetaine, which is assumed to be of no toxicological concern and is excreted unchanged in urine [27]. According to a risk assessment conducted by European Food Safety Authority (EFSA), Norway was considered the country with highest intake of arsenic in Europe, due to the high consumption of fish [27]. In Norway, foods with high concentration of inorganic arsenic are rice and rice products, supplements based on algae, and shellfish [50]. However, cereals and cereal products, vegetables, bottled water, and coffee contribute with the highest intake because they are consumed more frequently or in higher volumes [28].

Our study adds important data on iodine nutrition and thyroid function in population groups consuming vegan, lacto-ovo vegetarian and pescatarian diets. A strength of this study was the sample size of over 200 participants, compared to previous studies in people following plant-based diets. Another strength is that we measured several indicators of thyroid function and iodine nutrition: thyroid hormones, thyroglobulin, iodine supplement, macroalgae and urinary iodine. Limitations of the study were: lack of reference values for thyroglobulin [28] and the non-randomized study design, which resulted in smaller sample size in the pescatarian groups. The study also lacked a control group, but we included pescatarian groups which include all the dietary iodine sources in Norway. However, it is still a limitation that we did not include an omnivore group, as pescatarians might differ from the general populations as they might limit the intake of dairy products, eggs, and seafood to more extent.

## Conclusions

The prevalence of subclinical hypothyroidism was low and fT4 and fT3 were within the normal range for all dietary groups. Vegans had significantly increased Tg compared to pescatarians. All dietary groups had UIC indicating mild-to-moderate iodine deficiency, with lowest UIC in vegans. No differences were found between dietary groups in selenium- and arsenic concentrations. Although a low iodine intake may not have an acute effect on thyroid function, over time it may increase the risk of thyroid disorders, thus individuals with low iodine intakes are recommended to consume dietary iodine supplements.

### Supplementary Information

Below is the link to the electronic supplementary material.

Supplementary file1 (DOCX 47 kb)

## Data Availability

The data presented in this study are available on request from the corresponding author.

## References

[1] Zimmermann BM (2011). The role of iodine in human growth and development. Semin Cell Dev Biol.

[2] Ittermann T, Albrecht D, Arohonka P, Bilek R, de Castro JJ, Dahl L (2020). Standardized map of iodine status in Europe. Thyroid.

[3] Groufh-Jacobsen S, Mosand LM, Bakken KS, Solvik BS, Oma I, Gjengedal ELF (2020). Mild to moderate iodine deficiency and inadequate iodine intake in lactating women in the inland area of Norway. Nutrients.

[4] Henjum S, Abel MH, Meltzer HM, Dahl L, Alexander J, Torheim LE (2019). Is iodine intake adequate in Norway?. Tidsskr Nor Laegeforen.

[5] WHO (2007) Assessment of iodine deficiency disorders and monitoring their elimination—a guide for programme managers. https://www.who.int/nutrition/publications/micronutrients/iodine_deficiency/9789241595827/en/

[6] Zimmermann MB, Andersson M (2012). Assessment of iodine nutrition in populations: past, present, and future. Nutr Rev.

[7] Ma ZF, Skeaff SA (2014). Thyroglobulin as a biomarker of iodine deficiency: a review. Thyroid.

[8] Citterio CE, Targovnik HM, Arvan P (2019). The role of thyroglobulin in thyroid hormonogenesis. Nat Rev Endocrinol.

[9] Neufingerl N, Eilander A (2021). Nutrient intake and status in adults consuming plant-based diets compared to meat-eaters: a systematic review. Nutrients.

[10] Aakre I, Tveito Evensen L, Kjellevold M, Dahl L, Henjum S, Alexander J (2020). Iodine status and thyroid function in a group of seaweed consumers in Norway. Nutrients.

[11] Aakre I, Solli DD, Markhus MW, Mæhre HK, Dahl L, Henjum S (2021). Commercially available kelp and seaweed products—valuable iodine source or risk of excess intake?. Food Nutr Res.

[12] Clegg ME, Tarrado Ribes A, Reynolds R, Kliem K, Stergiadis S (2021). A comparative assessment of the nutritional composition of dairy and plant-based dairy alternatives available for sale in the UK and the implications for consumers' dietary intakes. Food Res Int.

[13] Bath SC, Hill S, Infante HG, Elghul S, Nezianya CJ, Rayman MP (2017). Iodine concentration of milk-alternative drinks available in the UK in comparison with cows' milk. Br J Nutr.

[14] Winther KH, Rayman MP, Bonnema SJ, Hegedüs L (2020). Selenium in thyroid disorders—essential knowledge for clinicians. Nat Rev Endocrinol.

[15] Schomburg L, Mariotti F (2017). Plant-based diets and selenium intake and status. Vegetarian and Plant-based diets in health and disease prevention.

[16] Combs GF (2015). Biomarkers of selenium status. Nutrients.

[17] EFSA (2014). Scientific opinion on dietary reference values for selenium. EFSA J.

[18] Phiri FP, Ander EL, Lark RM, Bailey EH, Chilima B, Gondwe J (2020). Urine selenium concentration is a useful biomarker for assessing population level selenium status. Environ Int.

[19] Palazzolo DL, Jansen KP (2008). The minimal arsenic concentration required to inhibit the activity of thyroid peroxidase activity in vitro. Biol Trace Elem Res.

[20] Molin M, Ulven SM, Dahl L, Lundebye AK, Holck M, Alexander J (2017). Arsenic in seafood is associated with increased thyroid-stimulating hormone (TSH) in healthy volunteers—a randomized controlled trial. J Trace Elem Med Biol.

[21] Vahter M (2002). Mechanisms of arsenic biotransformation. Toxicology.

[22] Hughes MF (2006). Biomarkers of exposure: a case study with inorganic arsenic. Environ Health Perspect.

[23] Groufh-Jacobsen S, Hess SY, Aakre I, Folven Gjengedal EL, Blandhoel Pettersen K, Henjum S (2020). Vegans, vegetarians and pescatarians are at risk of iodine deficiency in Norway. Nutrients.

[24] ISO (2003) In vitro diagnostic medical devices—measurement of quantities in samples of biological origin—metrological traceability of values assigned to calibrators and control materials. https://www.iso.org/standard/69984.html

[25] Saravanabhavan G, Werry K, Walker M, Haines D, Malowany M, Khoury C (2017). Human biomonitoring reference values for metals and trace elements in blood and urine derived from the Canadian Health Measures Survey 2007–2013. Int J Hyg Environ Health.

[26] Hadrup N, Ravn-Haren G (2020). Acute human toxicity and mortality after selenium ingestion: a review. J Trace Elem Med Biol.

[27] EFSA (2009). Scientific opinion on arsenic in food. EFSA J.

[28] Andersson M, Hunziker S, Fingerhut R, Zimmermann MB, Herter-Aeberli I (2020). Effectiveness of increased salt iodine concentration on iodine status: trend analysis of cross-sectional national studies in Switzerland. Eur J Nutr.

[29] Kesteloot H, Joossens JV (1996). On the determinants of the creatinine clearance: a population study. J Hum Hypertens.

[30] Stinca S, Andersson M, Erhardt J, Zimmermann MB (2015). Development and validation of a new low-cost enzyme-linked immunoassay for serum and dried blood spot thyroglobulin. Thyroid.

[31] Zimmermann MB, Boelaert K (2015). Iodine deficiency and thyroid disorders. Lancet Diabetes Endocrinol.

[32] Krejbjerg A, Bjergved L, Bülow Pedersen I, Carlé A, Knudsen N, Perrild H (2016). Serum thyroglobulin as a biomarker of iodine deficiency in adult populations. Clin Endocrinol (Oxf).

[33] Laurberg P, Cerqueira C, Ovesen L, Rasmussen LB, Perrild H, Andersen S (2010). Iodine intake as a determinant of thyroid disorders in populations. Best Pract Res Clin Endocrinol Metab.

[34] Petersen M, Knudsen N, Carlé A, Andersen S, Jørgensen T, Perrild H (2019). Increased incidence rate of hypothyroidism after iodine fortification in Denmark: a 20-year prospective population-based study. J Clin Endocrinol Metab.

[35] Laurberg P, Jørgensen T, Perrild H, Ovesen L, Knudsen N, Pedersen IB (2006). The Danish investigation on iodine intake and thyroid disease, DanThyr: status and perspectives. Eur J Endocrinol.

[36] Petersen M, Bülow Pedersen I, Knudsen N, Andersen S, Jørgensen T, Perrild H (2019). Changes in subtypes of overt thyrotoxicosis and hypothyroidism following iodine fortification. Clin Endocrinol (Oxf).

[37] Tonstad S, Nathan E, Oda K, Fraser G (2013). Vegan diets and hypothyroidism. Nutrients.

[38] Tonstad S, Jaceldo-Siegl K, Messina M, Haddad E, Fraser GE (2016). The association between soya consumption and serum thyroid-stimulating hormone concentrations in the Adventist Health Study-2. Public Health Nutr.

[39] Leung AM, Lamar A, He X, Braverman LE, Pearce EN (2011). Iodine status and thyroid function of Boston-area vegetarians and vegans. J Clin Endocrinol Metab.

[40] Key T, J., A, Thorogood M, Keenan J, Long A. (1992). Raised thyroid stimulating hormone associated with kelp intake in British vegan men. J Hum Nutr Diet.

[41] Rauma AL, Törmälä ML, Nenonen M, Hänninen O (1994). Iodine status in vegans consuming a living food diet. Nutr Res.

[42] Abdulla M, Andersson I, Asp NG, Berthelsen K, Birkhed D, Dencker I (1981). Nutrient intake and health status of vegans. Chemical analyses of diets using the duplicate portion sampling technique. Am J Clin Nutr.

[43] Delanghe J, De Slypere JP, De Buyzere M, Robbrecht J, Wieme R, Vermeulen A (1989). Normal reference values for creatine, creatinine, and carnitine are lower in vegetarians. Clin Chem.

[44] Hoet P, Jacquerye C, Deumer G, Lison D, Haufroid V (2013). Reference values and upper reference limits for 26 trace elements in the urine of adults living in Belgium. Clin Chem Lab Med.

[45] White MA, Sabbioni E (1998). Trace element reference values in tissues from inhabitants of the European Union. X. A study of 13 elements in blood and urine of a United Kingdom population. Sci Total Environ.

[46] Fallon N, Dillon SA (2020). Low intakes of iodine and selenium amongst Vegan and Vegetarian women highlight a potential nutritional vulnerability. Front Nutr.

[47] Rayman MP, Rayman MP (2002). The argument for increasing selenium intake. Proc Nutr Soc.

[48] VKM (2022) Benefit and risk assessment of fish in the Norwegian diet

[49] Birgisdottir BE, Knutsen HK, Haugen M, Gjelstad IM, Jenssen MTS, Ellingsen DG (2013). Essential and toxic element concentrations in blood and urine and their associations with diet: Results from a Norwegian population study including high-consumers of seafood and game. Sci Total Environ.

[50] VKM (2016) Dietary exposure to inorganic arsenic in the Norwegian population

