# Supplementary material for: Thyroid function and urinary concentrations of iodine, selenium, and arsenic in vegans, lacto-ovo vegetarians and pescatarians
Source: Eur J Nutr. 2023 Aug 17;62(8):3329–38. doi: 10.1007/s00394-023-03218-5 (PMC10611878; doi:10.1007/s00394-023-03218-5)
Supplement: Supplementary file 1 — Supplementary file1 (DOCX 47 kb) [file 394_2023_3218_MOESM1_ESM.docx]

**Supplemental Table 1**. Definitions of thyroid dysfunction and TPO-Ab positive individuals

| **Thyroid dysfunction** | **Reference range** |  |
| --- | --- | --- |
| Subclinical hypothyroidism | S-TSH >4.0 mU/L and S-fT4 within 11.0-23.0 pmol/L | |
| Overt hypothyroidism | S-TSH >4.0 mU/L and S-fT4 <11.0 pmol/L | |
| Isolated hypothyroxinemia | S-fT4 <11.0 pmol/L and S-TSH within 0.20-4.0 mU/L | |
| Subclinical hyperthyroidism | S-TSH <0.20 mU/L and S-fT4 within 11.0-23.0 pmol/L | |
| Overt hyperthyroidism | S-TSH <0.20 mU/L and S-fT4 >23.0 pmol/L | |
| Peroxidase antibody (anti-TPO) positivity | S-Anti-TPO >100 kU/L | |

TSH=Serum stimulating thyroid hormone; S-fT4=free thyroxine; S-Anti-TPO=serum anti-TPO
